# The C-Terminal Transactivation Domain of STAT1 Has a Gene-Specific Role in Transactivation and Cofactor Recruitment

**DOI:** 10.3389/fimmu.2018.02879

**Published:** 2018-12-06

**Authors:** Matthias Parrini, Katrin Meissl, Mojoyinola Joanna Ola, Therese Lederer, Ana Puga, Sebastian Wienerroither, Pavel Kovarik, Thomas Decker, Mathias Müller, Birgit Strobl

**Affiliations:** ^1^Department of Biomedical Sciences, Institute of Animal Breeding and Genetics, University of Veterinary Medicine Vienna, Vienna, Austria; ^2^Max F. Perutz Laboratories, University of Vienna, Vienna, Austria; ^3^University Center Biomodels Austria, University of Veterinary Medicine Vienna, Vienna, Austria

**Keywords:** macrophage, IFNγ, interferon regulatory factor 1 (IRF1), IRF8, transcriptional coactivator, mediator, RNA polymerase II, signal transducer and activator of transcription

## Abstract

STAT1 has a key role in the regulation of innate and adaptive immunity by inducing transcriptional changes in response to cytokines, such as all types of interferons (IFN). STAT1 exist as two splice isoforms, which differ in regard to the C-terminal transactivation domain (TAD). STAT1β lacks the C-terminal TAD and has been previously reported to be a weaker transcriptional activator than STAT1α, although this was strongly dependent on the target gene. The mechanism of this context-dependent effects remained unclear. By using macrophages from mice that only express STAT1β, we investigated the role of the C-terminal TAD during the distinct steps of transcriptional activation of selected target genes in response to IFNγ. We show that the STAT1 C-terminal TAD is absolutely required for the recruitment of RNA polymerase II (Pol II) and for the establishment of active histone marks at the class II major histocompatibility complex transactivator (*CIIta*) promoter IV, whereas it is dispensable for histone acetylation at the guanylate binding protein 2 (*Gbp2*) promoter but required for an efficient recruitment of Pol II, which correlated with a strongly reduced, but not absent, transcriptional activity. IFNγ-induced expression of *Irf7*, which is mediated by STAT1 in complex with STAT2 and IRF9, did not rely on the presence of the C-terminal TAD of STAT1. Moreover, we show for the first time that the STAT1 C-terminal TAD is required for an efficient recruitment of components of the core Mediator complex to the IFN regulatory factor (*Irf*) 1 and *Irf8* promoters, which both harbor an open chromatin state under basal conditions. Our study identified novel functions of the STAT1 C-terminal TAD in transcriptional activation and provides mechanistic explanations for the gene-specific transcriptional activity of STAT1β.

## Introduction

Signal-induced reprogramming of gene expression is a crucial part of cellular responses to environmental stimuli. Inducible transcriptional control relies on signal-activated transcription factors (TFs) that bind to DNA regulatory elements distant from the transcriptional start site (TSS) and facilitate the recruitment of transcriptional co-regulators and the general transcriptional machinery, including RNA polymerase II (Pol II). Binding to co-regulatory proteins, such as chromatin remodeling and histone modifying enzymes, occurs through one or more transactivation domains (TADs, also called activation domains) present in TFs (1, 2). Mediator, a large modular protein complex with varying subunit composition, bridges TFs with Pol II and coordinates DNA-loop formation, transcriptional initiation, and post-initiation events (3, 4). Transcription can be induced by *de novo* recruitment of Pol II, which requires assembly of a pre-initiation complex (PIC), or by releasing Pol II from a paused state into productive elongation (5–8). Transcriptional induction is accompanied by phosphorylation of Pol II at serine (S) residues in the heptapeptide repeats within its C-terminal domain (CTD). S5 phosphorylation is triggered by cyclin dependent kinase (CDK) 7, the kinase subunit of the general TF (GTF) complex TFIIH, and allows Pol II to initiate transcription. Typically, after 20–60 nucleotides from the TSS, Pol II is driven into a paused condition by negative elongation factors. S2 phosphorylation of the Pol II CTD is executed by CDK9, the kinase subunit of the positive transcription elongation factor b (p-TEFb), which also phosphorylates negative elongation factors and enables the release of paused Pol II from the promoter (9).

Signal transducer and activator of transcription (STAT) 1 is used for signaling by several cytokines, including all types of IFNs, which are crucial regulators of innate and adaptive immunity. Absence of STAT1 in humans and mice results in severe immunodeficiencies, including high sensitivity to bacterial and viral infections (10, 11). Activation of STAT1 occurs through phosphorylation at tyrosine 701 (Y701) by receptor-associated Janus kinases (JAKs). Type II IFN (IFNγ) mainly activates STAT1 homodimers, which translocate to the nucleus and bind to gamma-IFN activated sequences (GAS) in target gene promoters. Type I and type III IFNs mainly signal through the IFN-stimulated gene factor 3 (ISGF3) TF complex, which consists of STAT1, STAT2, and IFN regulatory factor 9 (IRF9), and binds to IFN-stimulated response elements (ISRE) (11, 12). The STAT1 TAD has been initially identified by the characterization of the naturally occurring splice variants STAT1α and STAT1β. The latter lacks 38 amino acids at the C-terminus and was unable to induce transcription in response to IFNγ when transfected into STAT1-deficient cells and analyzed *in vitro* using chromatin templates (13, 14). Moreover, transactivating activity could be transferred by fusing the 39 C-terminal amino acids to the yeast GAL4 DNA-binding domain (15–17). The STAT1 C-terminal TAD is constitutively active but its function can be modulated by phosphorylation at S727 (18, 19). In the context of IFNγ, S727 phosphorylation occurs within chromatin and is mediated by CDK8 (18). The probably best described function of the C-terminal TAD of STAT1 is its interaction with the histone acetyltransferase CBP/p300 (20, 21). The STAT1 C-terminal TAD also directly interacts with minichromosome maintenance protein 5 (MCM5) and DNA repair-associated tumor suppressor BRCA1 (17, 22, 23). However, the N-terminal region of STAT1 can also bind p300/CBP (24) and it remained unclear whether regions distinct from the C-terminal TAD contribute to the interactions with MCM5 or BRCA1. Our studies with gene-modified mice have shown that the absence of the C-terminal TAD of STAT1 does not abolish transcriptional responses to IFNγ but has modest to severe effects on a subset of target genes (25). Deletion of the C-terminal TAD of STAT1 and mutation of S727 to alanine (S727A) have overlapping but not identical consequences on transcriptional responses to IFNγ (18, 19, 25), indicating that the functions of the C-terminal TAD are not solely exerted through its serine phosphorylation.

In this study we investigated the role of the STAT1 C-terminal TAD in transactivation and cofactor recruitment to paradigmatic IFNγ-inducible genes. The availability of mice that express only the STAT1β isoform (*Stat1*^β/β^) enabled us to analyze transcriptional activity of STAT1β in primary immune cells under control of the endogenous promoter (25). We report an essential role of the STAT1 C-terminal TAD for an efficient recruitment of distinct Mediator subunits to the *Irf1* and the *Irf8* gene promoters in primary macrophages and for the post-recruitment regulation of Pol II. We furthermore report that the STAT1 C-terminal TAD is absolutely required for the induction of class II major histocompatibility complex transactivator (*CIIta*) through enabling recruitment of Pol II, strongly promotes Pol II recruitment to the guanylate binding protein 2 (*Gbp2*) promoter but is dispensable for the ISRE-driven induction of *Irf7*. Our results shed new light on the communication of STAT1 with the transcriptional machinery and provide mechanistic insights into STAT1 isoform-specific transcriptional activities.

## Materials and Methods

### Mice and Ethics Statement

C57BL/6N (wild-type, *WT*) mice were purchased from Janvier Labs. *Stat1*^β/β^ (25), *Stat2*^−/−^ (26), *Irf9*^−/−^ (27), and *Irf1*^−/−^ (28) were on C57BL/6 background*. Stat1*^β/β^*Stat2*^−/−^ and *Stat1*^β/β^*Irf9*^−/−^ were generated by crossing *Stat1*^β/β^ with *Stat2*^−/−^ or *Irf9*^−/−^ mice. Mice were housed under specific pathogen-free conditions according to Federation of European Laboratory Animal Science Associations (FELASA) guidelines. Mice were bred at the University of Veterinary Medicine Vienna according to the guidelines of the Federal Ministry of Science, Research and Economy section 8ff of the Animal Science and Experiments Act, Tierversuchsgesetz [TVG], BMWF-68.205/0068-WF/V/3b/2015. The study did not involve animal experiments as defined in the TVG and did not require ethical approval according to the local and national guidelines.

### Cell Culture and Cytokines

Bone marrow-derived macrophages (BMDMs) were isolated and differentiated from bone marrow (tibia and femur) of 8–12 weeks old sex-matched mice. BMDMs were differentiated for 7–9 days on Petri dishes (Greiner Bio-One) in DMEM (Sigma-Aldrich) supplemented with 10% FCS (Gibco/Thermo Fisher Scientific), 15% L929 cell-conditioned medium, 2 mM L-glutamine (Sigma-Aldrich), 100 U/ml penicillin, 100 μg/ml streptomycin, (Sigma-Aldrich) and 50 μM β-mercaptoethanol (Gibco/Thermo Fisher Scientific). Cells were treated with recombinant mouse 100 U/ml IFNγ (Millipore, IF005) for the times indicated.

### mRNA and Pre-mRNA Expression Analysis

Total RNA was isolated using peqGOLD TriFast^TM^ (VWR) according to manufacturer's instructions. cDNA synthesis and RT-qPCR were performed as described (25, 29). For assays that are located in introns or exon-intron junctions, total RNA was DNase-treated prior to cDNA synthesis. Controls without reverse transcriptase were included for all RT-qPCRs. Primers for pre-mRNA analyses are listed in Supplementary Table 1. Sequences of primers and probes for *Ube2d2* (ID 3377) and *Irf1* (ID 3848) mRNA analysis are available at the Real-Time Primer and Probe Database (http://www.rtprimerdb.org/). Primers for *Irf7* (QT00245266) and *Irf8* (QT00174195) mRNA analysis were purchased from Qiagen. qPCRs were done in duplicate on a Bio-Rad CFX96 Touch™ realtime machine.

### Whole Cell Extracts and Western Blotting

BMDMs (10^6^ cells/well) were stimulated with IFNγ (100 U/ml) for the times indicated, lysed and used for Western blot analysis as described previously (30) with the following adaptations: cells were lysed in 50 mM Tris-HCl (pH 8.0), 150 mM NaCl, 0.5% IGEPAL CA-630 (v/v), 10% glycerol (v/v), 0.1 mM EDTA, 2 mM DTT, 0.2 mM Na-vanadate, 25 mM Na-fluoride, 1 μg/ml leupeptin, 1 μg/ml aprotinin, 0.1 μg pepstatin and 1 mM PMSF. The following antibodies were used: anti-IRF1 (Santa Cruz, SC-640), anti-phospho-Tyr701 STAT1, and anti-STAT1 (Cell Signaling Technology, 9167 and 9172), anti-pan-ERK (BD Transduction Laboratories, 610123; p42 is shown in our experiments). Peroxidase-conjugated secondary antibodies (mouse and rabbit) were from Cell Signaling Technology (7076 and 7074). Blots were scanned with a Chemidoc analyzer (Bio-Rad).

### Flow Cytometric Analysis of MHC Class II

BMDMs were stimulated with 100 U/mL of IFNγ for 24 h, washed with PBS, harvested and stained for 15 min at 4°C with anti-MHC Class II (I-A/I-E)-PE (BD Biosciences, BD-557000) or isotype control (rat IgG2bκ-PE, BD Biosciences, BD553989). Data were acquired on a BD FACSCanto II and analyzed with the BD FACSDiva software version 8 (BD Biosciences).

### Chromatin Immunoprecipitation (ChIP) Assay and qPCR

The ChIP protocol was adapted from Nissen and Yamamoto (31) and Hauser et al. (32) with the following modifications: 2.5 × 10^7^ cells were cross-linked for 10 min at room temperature with 1% formaldehyde in PBS. For H3, H3ac, H4ac, H3K4me3, Pol II, S5pPol II, S2pPol II, STAT1, STAT3, and CDK9 ChIPs, cells were lysed with wash buffer I and II as described (32) and nuclei were lysed in 50 mM Tris-HCl pH 8, 1% SDS, 10 mM EDTA, 1x SIGMAFAST™ Protease Inhibitor (Sigma-Aldrich) and 1 mM PMSF. For STAT2, MED1, MED4, MED18, MED24, MED26, and ERCC3 ChIPs, cells were lysed as described (31) in 10 mM Tris-HCl at pH 8, 1 mM EDTA, 0.5 mM EGTA, 100 mM NaCl, 0.1% Na-deoxycholate, 0.5% N-lauroylsarcosine, 1x SIGMAFAST™ Protease Inhibitor and 1 mM PMSF. For all ChIPs, 25 μg chromatin per IP was used. Antibodies were pulled down with 50 μl of Protein G Dynabeads® (30 mg/ml, Novex, 10009D). qPCRs were done in duplicate on a Stratagene MX3000 or a Bio-Rad CFX96 Touch™ qPCR machine. Primers are listed in Supplementary Table 2, primers for the *Irf1* and *Gbp2* gene bodies were as previously described (18). Values are displayed as % input control (for Pol II, S5pPol II, S2pPol II, STAT1, STAT2, MED1, MED4, MED18, MED24, MED26, ERCC3, and CDK9) or relative to H3 (H3ac, H4ac, and H3K4me3). The following antibodies were used: anti-STAT1 (Cell Signaling Technology, 9172, 5 μl/ChIP), anti-Pol2 (Santa Cruz Biotechnology, SC-899-X; 4 μg/ChIP), anti-S5pPol2 (Bethyl, A300-655A; 0.7 μg/ChIP), anti-S2pPol2 (Bethyl, A300-654A; 0.7 μg/ChIP), anti-MED1 (TRAP220, Santa Cruz Biotechnology, SC-5334-X; 4 μg/ChIP, anti-MED4 (Abcam, ab129170; 5 μl/ChIP), anti-MED18 (Bethyl, A300-777A; 0.7 μg/ChIP), anti-MED24 (TRAP100, Bethyl, A300-472A; 0.7 μg/ChIP), anti-MED26 (CRSP70, Santa Cruz Biotechnology, SC-48776-X; 4 μg/ChIP), anti-CDK9 (Santa Cruz Biotechnology, SC-484; 4 μg/ChIP), and anti-ERCC3 (TFIIH subunit, Bethyl, A301-337A; 0.7 μg/ChIP) antibody.

### Statistical Analysis

**Figure 1:**
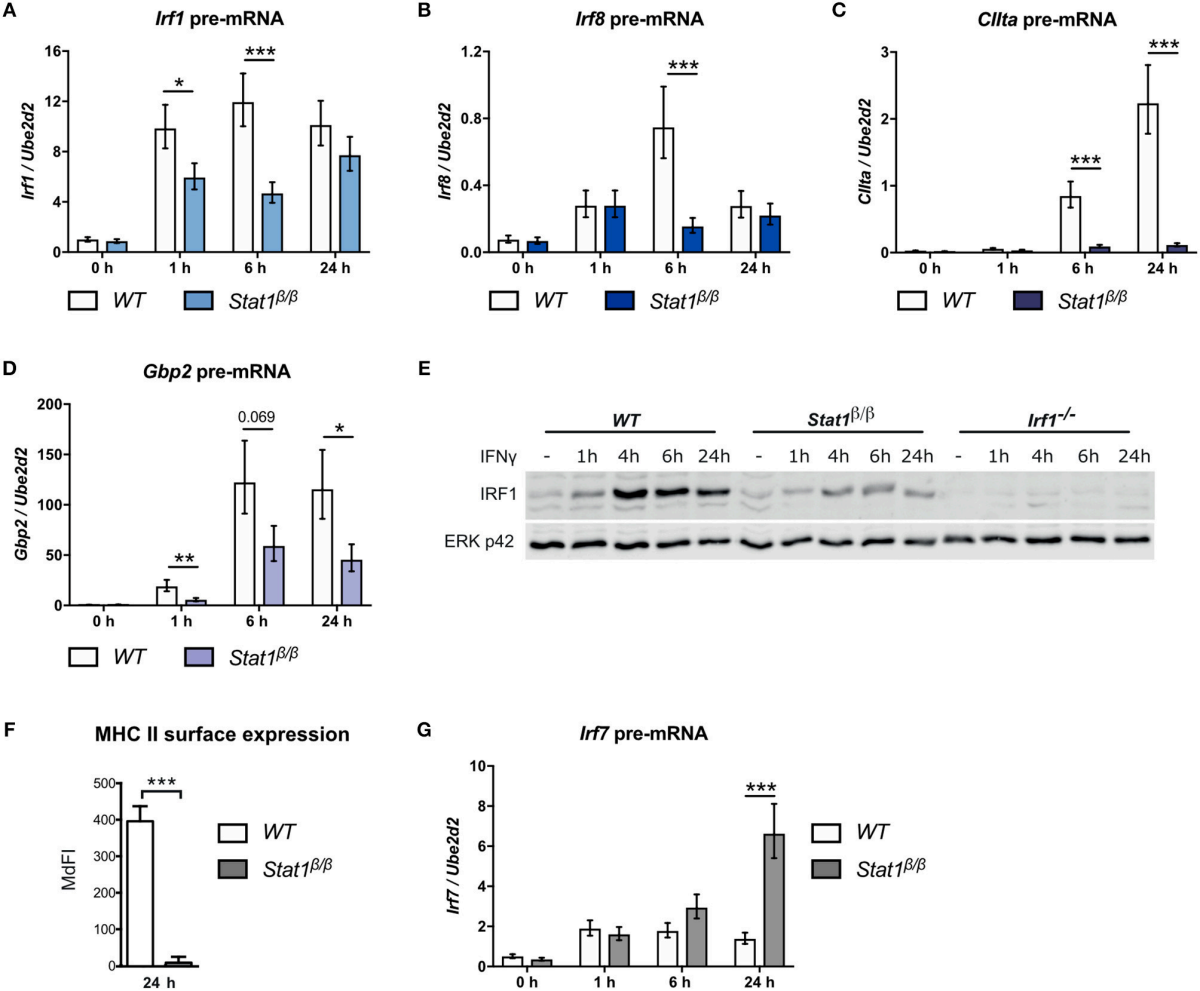
Transcriptional activity of STAT1β at the *Irf1, Irf8, CIIta, Gbp2*, and *Irf7* genes. BMDMs from *WT* and *Stat1*^β/β^ mice were stimulated with IFNγ for the times indicated or left untreated (0 h, -). Total RNA **(A–D, G)** or protein extracts **(E)** were isolated. *Irf1* **(A)***, Irf8* **(B)***, CIIta* **(C)***, Gbp2* **(D)**, and *Irf7* **(G)** pre-mRNA expression was determined by RT-qPCR. Data were normalized to the housekeeping gene *Ube2d2*. Mean values ± SE from three independent experiments are shown. **(E)** IRF1 protein levels were determined by Western blotting. ERK p42 was used as loading control. Data are as representative of two independent experiments. Original uncropped blots are shown in Supplementary Figure 1. **(F)** BMDMs from *WT* and *Stat1*^β/β^ mice were stimulated with IFNγ for 24 h. MHC class II surface levels were determined by flow cytometry. Median fluorescence intensities (MdFI) ±standard error (SE) from two independent experiments are shown. **(A–D, F–G)** ^*^P ≤ *0.05*, ^**^P ≤ *0.01*, ^***^P ≤ *0.001*. Significances are only indicated for the comparisons between genotypes.

Statistical analyses were done with IBM SPSS Version 22 (univariat mixed model with genotype and stimulation as fixed effects and experiment as random effect) or GraphPad Prism Version 6 (Student's *t*-test; Figure 1F).

## Results

### STAT1β Has Target Gene-Specific Transcriptional Activity

We have shown previously that STAT1β has a differential ability to induce target gene expression in response to IFNγ (25). However, total mRNA analysis is strongly influenced by mRNA decay rates and does not necessarily reflect transcriptional activity. Moreover, STAT1β shows prolonged tyrosine phosphorylation and prolonged *Irf1* and *Gbp2* promoter occupancy in the absence of STAT1α, which may prolong transcriptional activity (25). We thus analyzed pre-mRNA expression of paradigmatic target genes at different time points after IFNγ treatment in *Stat1*^β/β^ and *WT* cells. As STAT1 homodimer-driven primary response genes we selected *Irf1* and *Irf8* (33, 34), as secondary response genes that require cooperation of STAT1 dimers with IRF1 we analyzed *CIIta* and *Gbp2* (35–39) and as IFNγ-activated ISGF3-driven gene, we selected *Irf7* (40–42).

*Irf1* pre-mRNA expression was rapidly induced in response to IFNγ and was around 2-fold lower in *Stat1*^β/β^ compared to *WT* cells at 1 hour (h) and 6 h after treatment (Figure 1A). *Irf8* pre-mRNA expression was more transient and around 4-fold lower at 6 h after treatment in *Stat1*^β/β^ compared to *WT* cells (Figure 1B). Expression of both *Irf1* and *Irf8* pre-mRNAs did not differ between *Stat1*^β/β^ and *WT* cells at 24 h after treatment, suggesting that STAT1β does not show increased transcriptional activity at late time points after treatment (Figures 1A,B). As expected for secondary response genes, *CIIta* and *Gbp2* pre-mRNA synthesis increased at later time points after IFNγ treatment in *WT* cells (Figures 1C,D). *CIIta* pre-mRNA was barely detectable in *Stat1*^β/β^ cells (Figure 1C), whereas *Gbp2* pre-mRNA was clearly upregulated, albeit to reduced levels compared to *WT* cells (Figure 1D). In support of the pre-mRNA data, IFNγ induced considerably lower IRF1protein levels in *Stat1*^β/β^ than in *WT* cells (Figure 1E), whereas surface levels of the CIITA-regulated major histocompatibility complex class II (MHC II) proteins remained at basal levels in *Stat1*^β/β^ cells (Figure 1F). Surprisingly, *Irf7* pre-mRNA synthesis was profoundly increased 24 h after treatment in *Stat1*^β/β^ compared to *WT* cells, while it did not differ between the genotypes at early time points (Figure 1G). Taken together, these data show that STAT1β has gene-specific transcriptional activity which ranges from completely impaired (*CIIta*) or reduced (*Irf1, Irf8, Gbp2*) to an increased activity at late time points after IFNγ treatment (*Irf7*).

### Absence of STAT1α Differentially Impairs IFNγ-Induced Histone Modification and the Recruitment of Pol II to the *CIIta* and *Gbp2* Promoters

**Figure 2:**
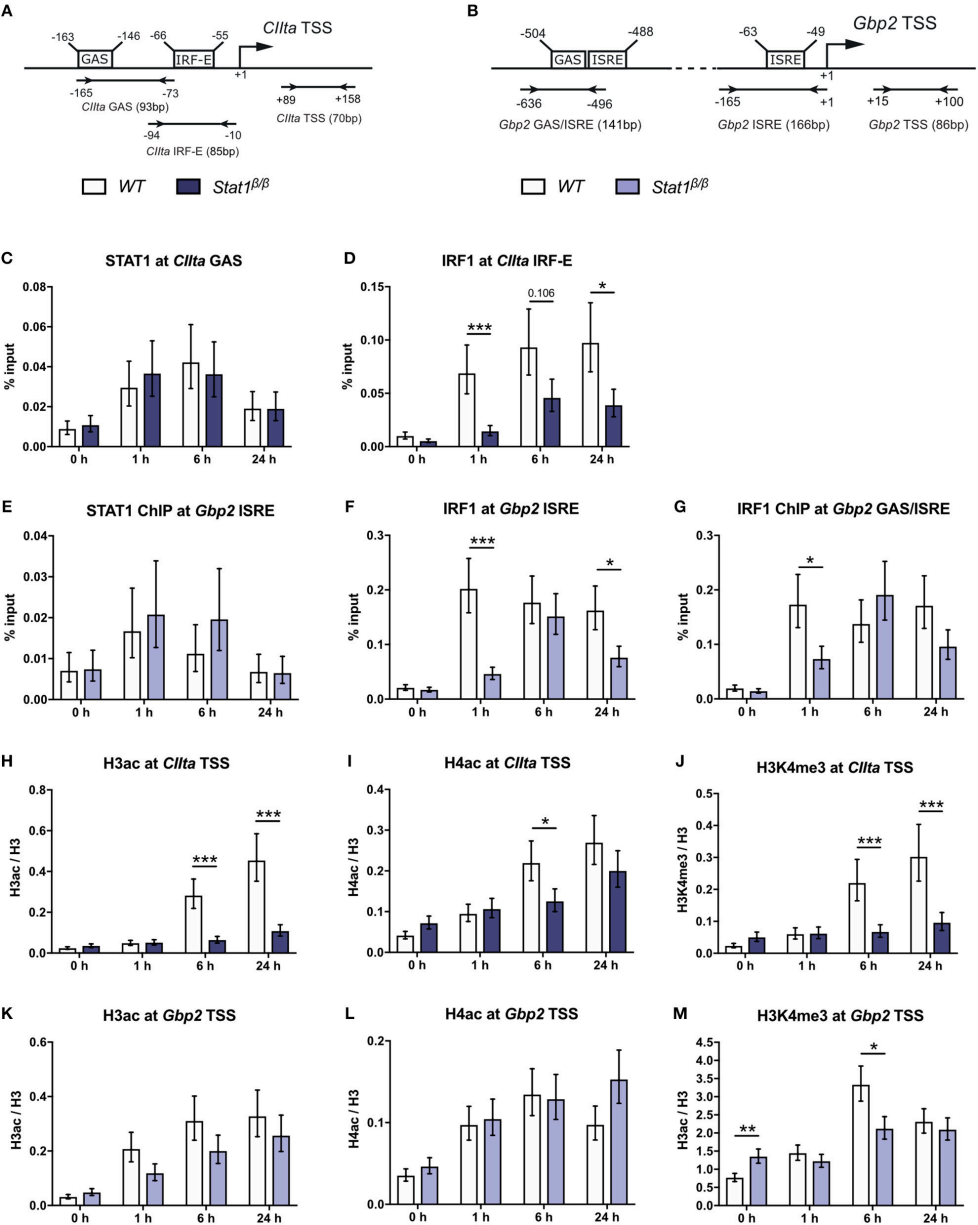
STAT1 and IRF1 binding to the *CIIta* promoter IV (pIV) and the *Gbp2* promoters and IFNγ-induced histone modifications. **(A,B)** Schematic representation of the murine *CIIta* and *Gbp2* promoter regions. STAT1 and IRF1 binding sites, the TSS, and the position of the primers used for the ChIP analyses are depicted.**(C–M)** BMDMs from *WT* and *Stat1*^β/β^ mice were stimulated with IFNγ for the times indicated or left untreated (0 h). STAT1 and IRF1 binding to the *CIIta* **(C, D)** and the *Gbp2* **(E–G)** promoter binding sites was analyzed by ChIP. H3 pan-acetylation (H3ac), H4 pan-acetylation (H4ac), and H3 lysine 4 trimethylation (H3K4me3) around the *CIIta* **(H–J)** and the *Gbp2* **(K–M)** TSS was determined by ChIP. Data were normalized to the input control **(C–G)** and the total levels of H3 **(H–M)**. Mean values ± SE from three **(C–G**, **H,J,K,M)** or four **(I,L)** independent experiments are shown; ^*^P ≤ *0.05*, ^**^P ≤ *0.01*, ^***^P ≤ *0.001*. Significances are only indicated for the comparisons between genotypes.

To test whether differences in *CIIta* and *Gbp2* expression relate to differences in STAT1 or IRF1 binding, we performed site-directed ChIP experiments. Transcriptional induction of *CIIta* in response to IFNγ requires chromatin remodeling by the SWI/SNF protein Brahma-related gene 1 (BRG1) (43). The presence of BRG1 is also required for STAT1 binding to the IFNγ-responsive *CIIta* promoter IV (pIV) and the *Gbp2* promoter (37). At the *CIIta* pIV, STAT1 binding additionally relies on cooperation with upstream transcription factor 1 (USF-1), which associates with the adjacent IRF-E box that is also bound by IRF1 (Figure 2A). STAT1 occupancy at the GAS site of the *CIIta* pIV was similar between *Stat1*^β/β^ and WT cells (Figure 2C), demonstrating that the STAT1 C-terminal TAD is not required for binding to the *CIIta* promoter and supporting previous studies demonstrating that the STAT1-BRG1 interaction is mediated through the N-terminal and coiled-coil domains of STAT1 (44). Despite the strongly reduced availability of IRF1 in *Stat1*^β/β^ cells, IRF1 was still detectable at *CIIta* pIV, although its binding was delayed and promoter occupancy was around 2–3-fold lower than in *WT* cells at 6 h after treatment (Figure 2D). The *Gbp2* promoter contains two IFNγ-responsive elements: a promoter proximal region containing an ISRE site and a distal region with adjoining GAS and ISRE sites (Figure 2B). The distal GAS site binds STAT1 dimers (38) and showed prolonged association with STAT1β in the absence of STAT1α (25). In contrast, the ISRE-containing proximal promoter binds non-canonical STAT1-containing complexes (19) and showed similar STAT1 occupancy in *Stat1*^β/β^ and *WT* cells (Figure 2E). In line with previous studies (45) we found association of IRF1 with the proximal and distal *Gbp2* promoter elements. Association of IRF1 with both promoter elements was delayed and reduced in *Stat1*^β/β^ compared to *WT* cells (Figures 2F,G). Taken together these data support previous studies indicating that the C-terminal TAD of STAT1 is not required for binding to GAS elements (21, 46, 47) and show that the reduced availability of IRF1 delays but does not completely abolish the recruitment of IRF1 to the *Gbp2* and *CIIta* promoters.

Transcriptional induction of *CIIta* and *Gbp2* by IFNγ is accompanied by an increase in acetylation of histones 3 and 4 (19, 37, 45, 48). IFNγ-induced histone 3 acetylation (H3ac) was nearly abolished and histone 4 acetylation (H4ac) strongly reduced around the *CIIta* pIV TSS in *Stat1*^β/β^ compared to *WT* cells (Figures 2H,I), whereas the upregulation of H3ac and H4ac at the *Gbp2* promoter was largely intact (Figures 2K,L). In contrast, IFNγ-induced H3 lysine 4 trimethylation (H3K4me3), which marks active promoter regions (49), was lower in *Stat1*^β/β^ cells at the *CIIta* and the *Gbp2* promoter (Figures 2J,M). *Stat1*^β/β^ cells had modestly higher levels of H3K4me3 at the *Gbp2* promoter than *WT* cells under basal conditions (Figure 2M), although this did not correlate with an increase in *Gbp2* pre-mRNA synthesis (Figure 1D).

**Figure 3:**
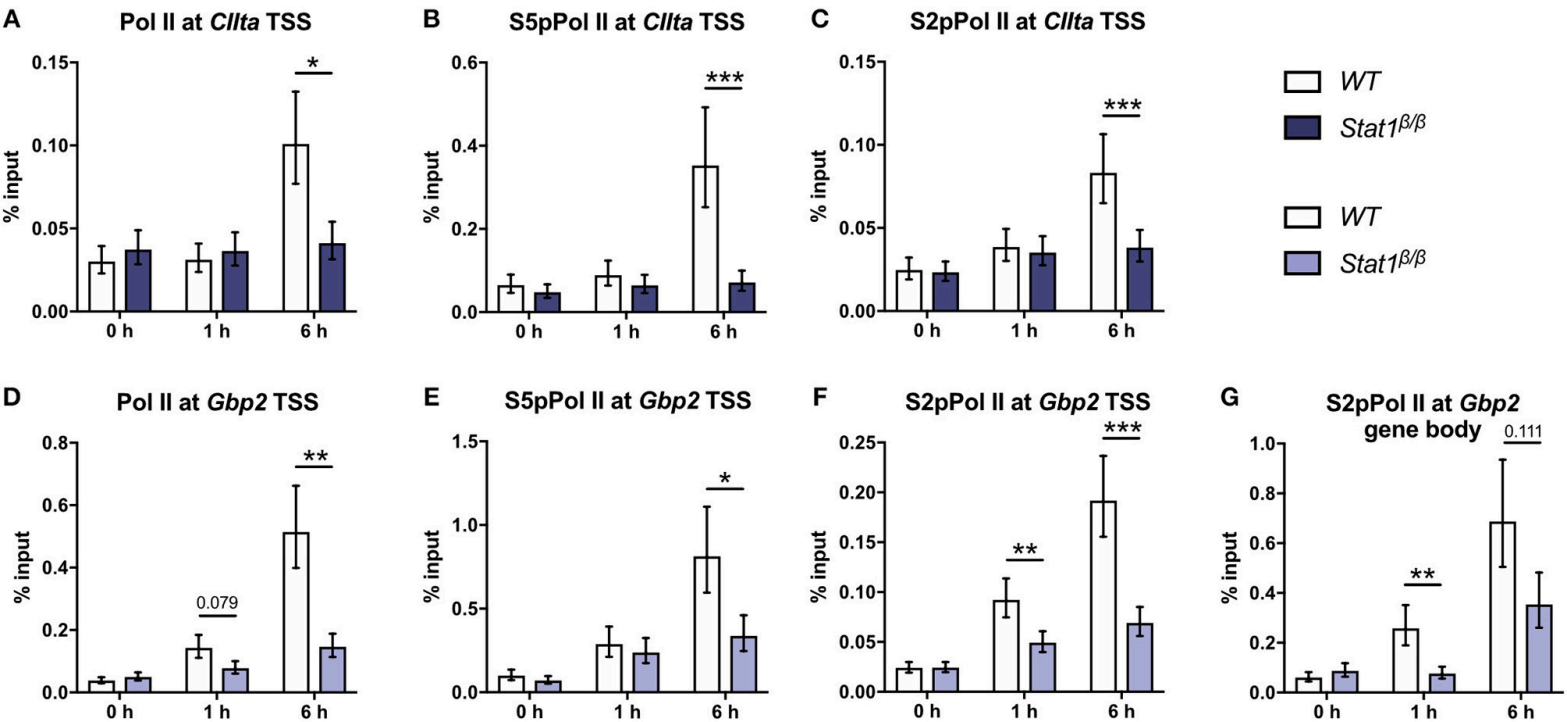
Promoter occupancy of Pol II, S5 phosphorylated Pol II (S5pPol II), and S2 phosphorylated Pol II (S2pPol II) around the *CIIta* and *Gbp2* TSS and of S2pPol II at the *Gbp2* gene body. BMDMs from *WT* and *Stat1*^β/β^ mice were stimulated with IFNγ for the times indicated or left untreated (0 h). The association of Pol II, S5pPol II, and S2pPol II with the TSS of **(A–C)** *CIIta* pIV and **(D–F)** *Gbp2* TSS, and **(G)** association of S2pPol II with the *Gbp2* gene body was determined by ChIP. Data were normalized to the input control. Mean values ± SE from three **(D,G)**, four **(A,B)** or five **(C,E,F)** independent experiments are shown; ^*^P ≤ *0.05*, ^**^P ≤ *0.01*, ^***^P ≤ *0.001*. Significances are only indicated for the comparisons between genotypes.

We next analyzed whether the differences in histone acetylation between *CIIta* and *Gbp2* in *Stat1*^β/β^ cells correlate with differences in the recruitment of Pol II. IFNγ induced a strong increase in Pol II occupancy at the *CIIta* pIV TSS at 6 h after treatment, which was completely absent in *Stat1*^β/β^ cells (Figure 3A). In line with the total Pol II data, promoter occupancy of S5 phosphorylated Pol II (S5pPol II) and S2pPol II did not increase around the *CIIta* TSS in *Stat1*^β/β^ cells in response to IFNγ (Figures 3B,C). Although Pol II recruitment and phosphorylation at the *Gbp2* promoter was also severely impaired in *Stat1*^β/β^ cells (Figures 3D–F), IFNγ still induced an increase in S2pPol II occupancy within the *Gbp2* gene body. In line with the pre-mRNA (Figure 1D), S2pPol II occupancy within the *Gbp2* gene body was strongly reduced in *Stat1*^β/β^ cells compared to *WT* cells (Figure 3G).

Taken together, these data indicate a differential requirement for the STAT1 C-terminal TAD for the establishment of active histone marks at the *CIIta* and *Gbp2* promoter and show that STAT1 C-terminal TAD-independent histone acetylation at the *Gbp2* is not sufficient to enable efficient recruitment Pol II.

### IRF9 and STAT2 Are Not Required for the Induction of *Irf1* by STAT1β

**Figure 4:**
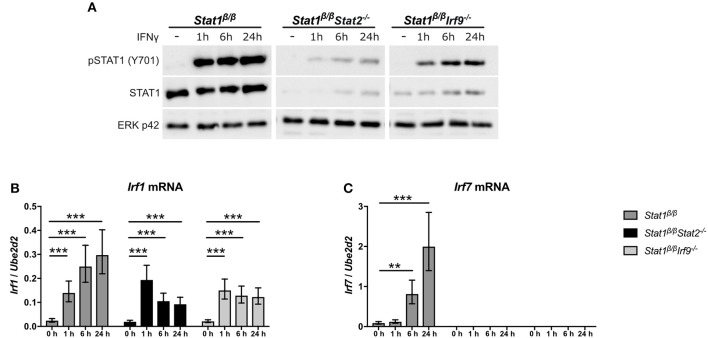
IFNγ induced expression of *Irf1* and *Irf7* in *Stat1*^β/β^ cells in the absence of STAT2 or IRF9. BMDMs derived from *Stat1*^β/β^*, Stat1*^β/β^*Stat2*^−/−^ and *Stat1*^β/β^*Irf9*^−/−^ mice were stimulated with IFNγ for the times indicated or left untreated (0 h, -). **(A)** Protein was isolated and Tyr701-phosphorylated STAT1 (pSTAT1) and STAT1 protein levels determined by Western blotting. ERK p42 was used as loading control. One representative out of three independent experiments is shown. Original uncropped blots are shown in Supplementary Figure 2. **(B,C)** Total RNA was isolated and *Irf1* **(B)** and *Irf7* **(C)** mRNA expression was determined by RT-qPCR. Data were normalized to *Ube2d2*. Mean values ± SE from three **(C)** or four **(B)** independent experiments are shown. ^*^P ≤ *0.05*, ^**^P ≤ *0.01*, ^***^P ≤ *0.001*. Significances are only indicated for the comparisons between genotypes.

It is becoming increasingly evident that IFNs not only signal through STAT1 homodimers and ISGF3 but also through non-canonical complexes, such as STAT1-STAT2 heterodimers, STAT1-IRF9, or STAT2-IRF9 (27, 50–52). To exclude that the absence of STAT1α favors the formation of non-canonical STAT1-complexes and to confirm that the induction of *Irf7* but not *Irf1* depends on the presence of STAT2 and IRF9, we crossed *Stat1*^β/β^ mice with mice lacking either STAT2 (*Stat1*^β/β^*Stat2*^−/−^) or IRF9 (*Stat1*^β/β^*Irf9*^−/−^). In line with the importance of type I IFN-ISGF3 signaling in the regulation of basal STAT1 expression (53), STAT1β protein levels were reduced in the absence of STAT2 and, to a lesser extent, in the absence of IRF9 (Figure 4A). However, *Irf1* mRNA was still upregulated in response to IFNγ in *Stat1*^β/β^*Stat2*^−/−^ and *Stat1*^β/β^*Irf9*^−/−^ cells (Figure 4B), confirming that its induction does not rely on the presence of IRF9 or STAT2. IFNγ-triggered *Irf7* mRNA expression was completely abolished in *Stat1*^β/β^*Stat2*^−/−^ and *Stat1*^β/β^*Irf9*^−/−^ cells (Figure 4C), supporting previous studies demonstrating that the induction of *Irf7* by IFNγ requires the presence of STAT2 and/or IRF9 (41). It is important to note that previous studies have established that other STAT proteins cannot compensate for the loss of STAT1 in upregulating most of the classical ISGs, including *Irf1* and *Irf8* (54–57), further underscoring the notion that STAT1β homodimers are capable of inducing GAS-driven genes, albeit to reduced levels as compared to STAT1α homodimers or STAT1α/STAT1β dimers.

### Absence of STAT1α Does Not Affect the Establishment of Active Histone Marks at the *Irf1* and *Irf8* Promoters

**Figure 5:**
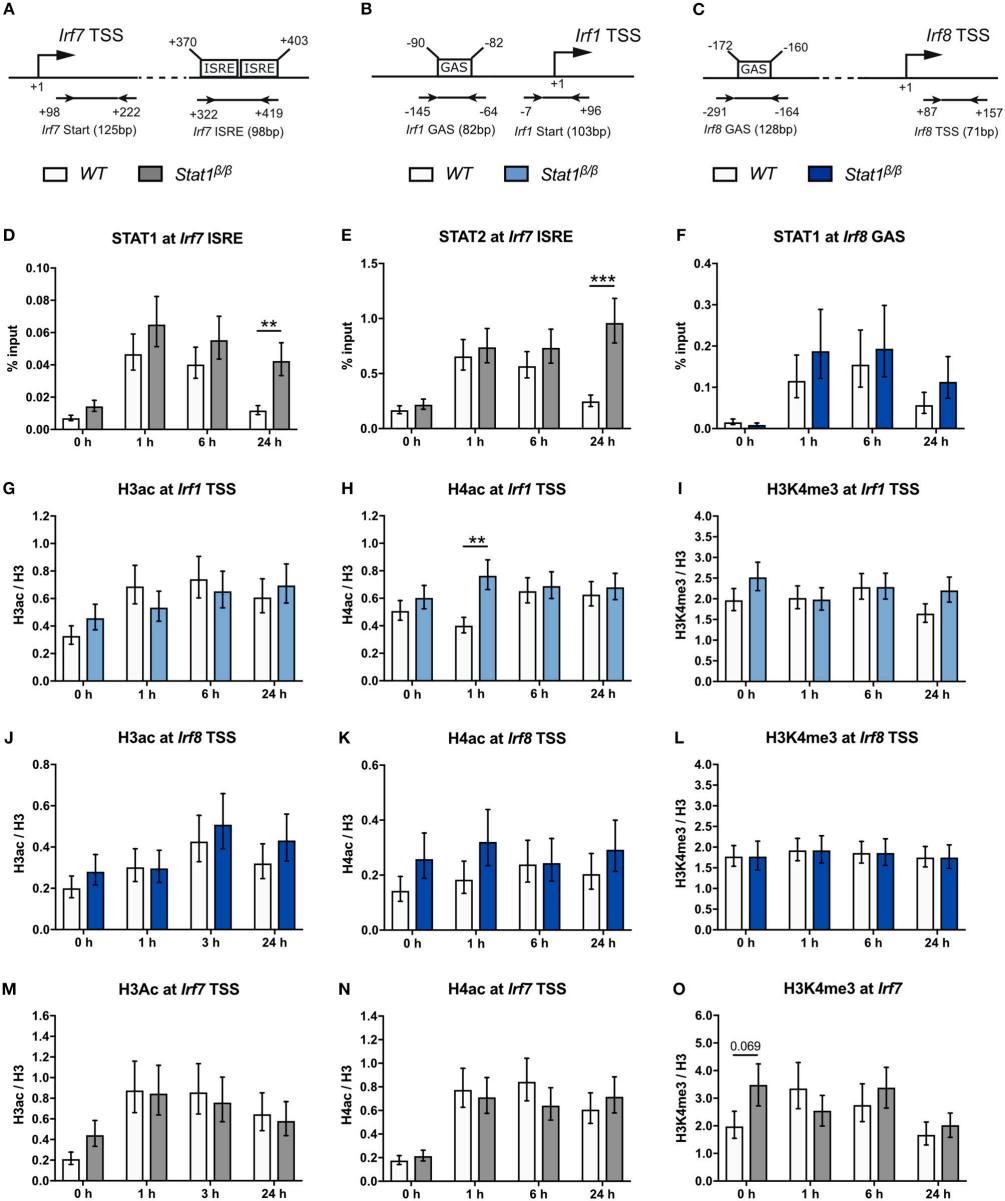
STAT1 and IRF1 binding to the *Irf7* and *Irf8* promoters and H3ac and H4ac and H3K4me3 around the *Irf1, Irf8*, and *Irf7* TSS before and after IFNγ treatment. **(A–C)** Schematic representation of the murine *Irf7* **(A)**, *Irf1* **(B)**, and *Irf8* **(C)** promoter regions. GAS and ISRE sites, the TSS and the position of the primers used for the ChIP analyses are depicted. **(D–O)** BMDMs from *WT* and *Stat1*^β/β^ mice were stimulated with IFNγ for the times indicated or left untreated (0 h). STAT1 and STAT2 binding at the *Irf7* ISRE **(D,E)** and STAT1 binding at the *Irf8* **(F)** GAS element was analyzed by ChIP. H3 pan-acetylation (H3ac), H4 pan-acetylation (H4ac), and H3 lysine 4 trimethylation (H3K4me3) around the *Irf1* **(G–I)**, the *Irf8* **(J–L)**, and the *Irf7* **(M–O)** TSS was determined by ChIP. Data were normalized to the input control **(D–F)** or the total levels of H3 **(G–O)**. Mean values ± SE from three to four independent experiments are shown; ^**^P ≤ *0.01*, ^***^P ≤ *0.001*. Significances are only indicated for the comparisons between genotypes.

We next investigated the impact of the STAT1 C-terminal TAD on STAT1 and STAT2 binding kinetics and the establishment of active histone marks at the *Irf1, Irf8*, and *Irf7* promoters. The *Irf7* promoter contains two adjoining ISRE sites downstream of its TSS (Figure 5A). Consistent with the regulation of *Irf7* by ISGF3 (41), IFNγ induced a rapid association of STAT1 and STAT2 to the *Irf7* promoter (Figures 5D,E). STAT1 and STAT2 occupancy was similar at 1 h and 6 h but considerably higher at 24 h after treatment in *Stat1*^β/β^ as compared to *WT* cells (Figures 5D,E). In contrast to our previous observations at the GAS sites of the *Irf1* promoter and the distal *Gbp2* promoter (25), STAT1 occupancy around the GAS site within the *Irf8* promoter was not different between *Stat1*^β/β^ and *WT* cells (Figure 5F), suggesting that the prolonged phosphorylation of STAT1β in the absence of STAT1α (25) prolongs promoter binding in a promoter context-specific manner.

In line with a previous study indicating that the *Irf1* gene harbors a permissive chromatin conformation under basal conditions in bone marrow-derived macrophages (58), we found higher H3ac, H4ac, and H3K4me3 to H3 ratios at the *Irf1* promotor than at the *CIIta* and *Gbp2* promoters in untreated cells, irrespective of the presence of STAT1α (Figures 5G–I, compare to Figures 2H–M). Except for an around 2-fold higher level of H4ac at 1 h after IFNγ treatment in *Stat1*^β/β^ cells, we did not observe differences between *Stat1*^β/β^ and *WT* cells (Figures 5G–I). Neither IFNγ treatment nor the absence of STAT1α affected the levels of H3ac, H4ac, or H3K4me3 at the *Irf8* promoter (Figures 5J–L). Despite the high basal H3ac and H4ac levels at the *Irf7* promoter, acetylation increased within 1 h of IFNγ treatment, which was again independent of the presence of the STAT1 C-terminal TAD (Figures 5M,N). In contrast, H3K4me3 levels did not increase in response to IFNγ treatment but slightly decreased at 24 h after treatment in *Stat1*^β/β^ and *WT* cells (Figure 5O).

### The C-Terminal TAD of STAT1 Facilitates an Efficient Recruitment of Mediator Complex Subunits to the *Irf1* Promoter and Promotes Transcription at a Post-Initiation Step

**Figure 6:**
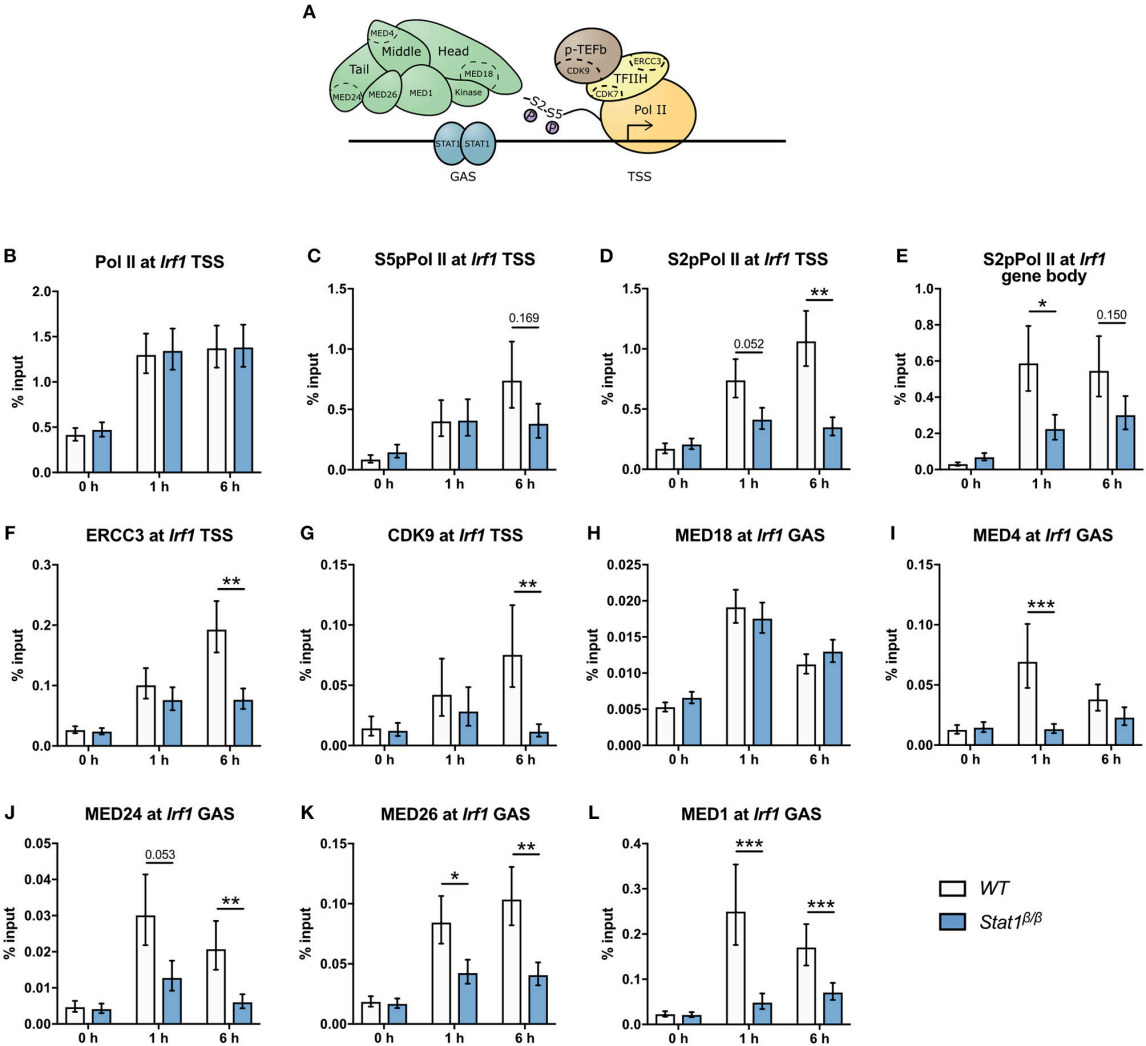
Recruitment and phosphorylation of Pol II at *Irf1*gene and occupancy of TFIIH, p-TEFb, and Mediator components at the *Irf1* TSS. **(A)** Schematic representation of Pol II, GTFs, and the Mediator complex at a GAS-driven gene promoter. Components of the Mediator, TFIIH, and p-TEFb complexes analyzed by ChIP are indicated. **(B–L)** BMDMs from *WT* and *Stat1*^β/β^ mice were stimulated with IFNγ for the times indicated or left untreated (0 h). Association of Pol II, S5pPol II, and S2pPol II with the *Irf1* promoter around the TSS **(B–D)** and of S2pPol II within the *Irf1* gene body **(E)**. Association of ERCC3 **(F)** and CDK9 **(G)** at the *Irf1* TSS and of MED18 **(H)**, MED4 **(I)**, MED24 **(J)**, MED26 **(K)**, and MED1 **(L)** at the *Irf1* GAS. Data were normalized to the input control. Mean values ± SE from two **(H)** or three (all others) independent experiments are shown; ^*^P ≤ *0.05*, ^**^P ≤ *0.01*, ^***^P ≤ *0.001*.

To address the question why STAT1β has reduced transcriptional activity at the *Irf1* gene we next analyzed the recruitment and phosphorylation of Pol II and the recruitment of components of the Mediator complex and GTF complexes TFIIH and p-TEFb (Figure 6A). IFNγ-induced an around 3-fold increase in Pol II promoter occupancy at the *Irf1* TSS in *Stat1*^β/β^ and *WT* cells (Figure 6B), indicating that the STAT1 C-terminal TAD is not required to recruit Pol II to the *Irf1* promoter. As shown in Figure 6C, the association of S5pPol II with the *Irf1* promoter increased upon IFNγ treatment and was not different between *Stat1*^β/β^ and *WT* cells at 1 h after treatment. Promoter occupancy of S5pPol II was modestly reduced at 6 h after treatment in *Stat1*^β/β^ compared to *WT* cells, although this did not reach statistical significance. CDK7, the kinase that phosphorylates Pol II at S5 in its CTD is a component of the TFIIH complex that also contains ERCC3 [(59) and Figure 6A). Consistent with the S5pPol II data, association of ERCC3 with the *Irf1* promoter was similar in *Stat1*^β/β^ and *WT* cells at 1 h, whereas it was reduced at 6 h after IFNγ treatment in *Stat1*^β/β^ compared to *WT* cells (Figure 6F). To proceed into productive elongation Pol II requires the recruitment of the p-TEFb complex and the activation of its associated kinase CDK9, which can phosphorylate Pol II at S2 in its CTD [(6) and Figure 6A). CDK9 promoter occupancy (Figure 6G) and association of S2pPol II with the *Irf1* promoter (Figure 6D) did not significantly differ between *Stat1*^β/β^ and *WT* cells 1 h after treatment but were strongly reduced at 6 h after treatment (Figures 6G,D). In contrast, levels of S2pPol II within the *Irf1* gene body, which is an indicator for productive transcriptional elongation, was already clearly lower at 1 h after treatment in *Stat1*^β/β^ than in *WT* cells (Figure 6E). Thus, during the early phases of the IFNγ response the impaired release of poised Pol II is not due to an impaired recruitment of TFIIH or p-TEFb to the *Irf1* promoter. The Mediator complex is a central transcriptional co-activator that bridges TFs with Pol II and is involved in the regulation of multiple steps of the transcriptional cycle, including the formation of a stable PIC, transcriptional elongation and transcriptional re-initiation (3, 60). Given the high complexity of Mediator, we analyzed the recruitment of selected subunits of the head, middle and tail modules [(61) and Figure 6A) to the *Irf1* promoter. We found a profound increase of MED18 (head), MED4 (middle) and MED24 (tail) promoter occupancy around the *Irf1* GAS after IFNγ stimulation in *WT* macrophages (Figures 6H–J). Recruitment of MED18 did not differ between *Stat1*^β/β^ and *WT* cells, whereas recruitment of MED4 and MED24 was reduced in *Stat1*^β/β^ cells (Figures 6H–J). The MED1 and MED26 subunits are not always associated with the Mediator complex but, dependent on the target gene, can be central to its functionality. MED1 has been described important for nuclear receptor interaction (62, 63) and MED26 to interact with the super elongation complex, which contains p-TEFb (64). Similar to MED4 and MED24, MED26 and MED1 were recruited less efficiently to the *Irf1* promoter in *Stat1*^β/β^ than in *WT* cells (Figures 6K,L). *Stat1*^β/β^ cells already showed reduced association of Mediator components at the time point when promoter occupancy of ERCC3 and CDK9 did not differ from *WT* cells (i.e., 1 h after treatment, Figures 6F,G), indicating that the recruitment of TFIIH and p-TEFb to the *Irf1* promoter is independent of an increase in promoter association of MED1, MED4, MED24, and MED26 at the *Irf1* gene at early time points after stimulation.

### The Importance of the C-Terminal TAD of STAT1 for an Efficient Recruitment of Mediator Components and the Release of Poised Pol II Extends to the *Irf8* Promoter

**Figure 7:**
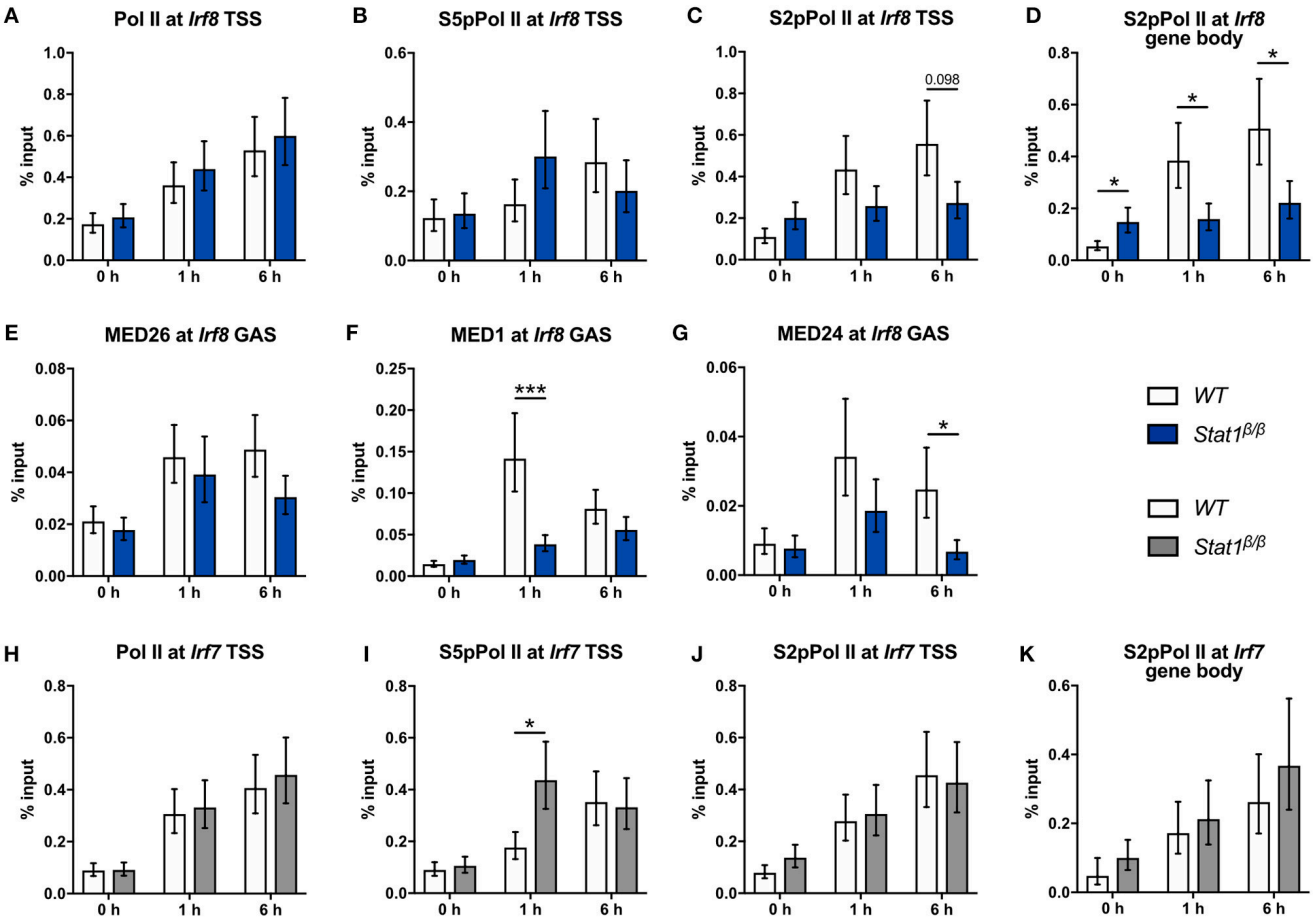
Recruitment and phosphorylation of Pol II at the *Irf8* and *Irf7* genes and occupancy of Mediator components at the *Irf8* promoter. **(A–K)** BMDMs from WT and *Stat1*^β/β^ mice were stimulated with IFNγ for the times indicated or left untreated (0 h). Association of Pol II, S5pPol II, and S2pPol II with the *Irf8* **(A–C)** and the *Irf7* promoters **(H–J)** around the TSS and of S2pPol II within the respective gene bodies **(D,K)** was determined by ChIP. The promoter occupancy of MED26 **(E)**, MED1 **(F)**, and MED24 **(G)** at the *Irf8* GAS. Data were normalized to the input control. Mean values ± SE from two **(K)**, three **(A–I)** or four **(J)** independent experiments are shown; ^*^P ≤ *0.05*, ^**^P ≤ *0.01*, ^***^P ≤ *0.001*.

Next, we analyzed the recruitment and phosphorylation of Pol II and the recruitment of Mediator components to the *Irf8* gene. IFNγ induced an around 2-fold increase in Pol II promoter occupancy at the *Irf8* TSS in *Stat1*^β/β^ and *WT* cells (Figure 7A). S5pPol II and S2pPol II occupancy at the *Irf8* promoter followed a similar pattern as at the *Irf1* promoter, although association of S2pPol II with the TSS was not significantly different between *Stat1*^β/β^ and *WT* cells after 6 h stimulation (Figures 7B,C). S2pPol II occupancy within the *Irf8* gene body was even higher in *Stat1*^β/β^ than in *WT* cells under basal conditions (Figure 7D), although this did not correlate with increased *Irf8* pre-mRNA levels (Figure 1B). In line with the pre-mRNA data, S2pPol II occupancy within the *Irf8* gene body was lower in *Stat1*^β/β^ than in *WT* cells at 1 and 6 h after treatment (Figure 7D). Taken together these data suggest that the STAT1 C-terminal TAD facilitates the release of Pol II into productive elongation also at the *Irf8* promoter. Although we were unable to reliably detect *Irf8* promoter sequences in MED4 and MED18 ChIPs under our experimental conditions, we observed reduced recruitment of MED1 and MED24, but not MED26, to the *Irf8* promoter in *Stat1*^β/β^ compared to *WT* cells (Figures 7E–G), indicating that the requirement for the C-terminal TAD of STAT1 for an efficient recruitment of subunits of the Mediator complex extends to other GAS-driven genes, such as *Irf8*, but may affect distinct Mediator subunits depending on the target gene.

In line with the unimpaired transcriptional induction of *Irf7* (Figure 1G), *Stat1*^β/β^ cells did not differ from *WT* cells with respect to the association of Pol II and S2pPol II at the *Irf7* TSS and S2pPol II within the *Irf7* gene body at 1 h and 6 h after IFNγ treatment (Figures 7H–K). Promoter occupancy of S5pPol II at the *Irf7* TSS was transiently higher in *Stat1*^β/β^ than in *WT* cells (Figure 7I) but this did not translate into higher levels of S2pPol II at the TSS or within the gene body or an increased transcriptional activity (Figures 7J,K and Figure 1G) at this time point. The finding that the induction of *Irf7* does not require the presence of the STAT1 C-terminal TAD is consistent with earlier studies indicating and that in the context of ISGF3 the TAD is provided by STAT2 (65).

## Discussion

In this study we used primary macrophages from mice that only express the STAT1β isoform to investigate the role of the C-terminal TAD of STAT1 in the IFNγ-induced transcriptional activation of the *Irf1, Irf7, Irf8, Gbp2*, and *CIIta* genes under physiologic conditions. Using pre-mRNA and ChIP analyses, we show for the first time that STAT1β has gene-specific transcriptional activity that correlates with a gene-specific requirement for the C-terminal TAD for IFNγ-induced histone modification, recruitment of Pol II and association of components of the Mediator complex to target gene promoters (Table 1).

**Table 1:** Summary of ChIP results at 1 and 6 h after IFNγ treatment.

**Gene**		***CIIta***		***Gbp2***		***Irf1***		***Irf8***		***Irf7***	
**Time of IFN**γ **treatment**		**1 h**	**6 h**	**1 h**	**6 h**	**1 h**	**6 h**	**1 h**	**6 h**	**1 h**	**6 h**
ChIP	site of PCR										
STAT1	GAS	=	=	↓^a^	=^a^	↓^a^	=^a^	=	=	NA	
STAT1	ISRE	NA		=	=	NA		NA		=	=^b^
STAT2	ISRE	NA		NA		NA		NA		=	=^b^
IRF1	ISRE/IRF-E	↓↓	=	↓↓	=	NA		NA		NA	
IRF1	GAS-ISRE	NA		↓↓	=	NA		NA		NA	
H3ac	TSS	–	↓↓	=	=	–	–	–	–	=	=
H4ac	TSS	–	↓	=	=	↑	–	–	–	=	=
H3K4me3	TSS	–	↓↓	–	↓	–	–	–	–	–	–
Pol II	TSS	–	↓↓	(↓)	↓↓	=	=	=	=	=	=
S5pPol II	TSS	–	↓↓	=	↓↓	=	=	–	=	↑	=
S2pPol II	TSS	–	↓↓	↓	↓↓	(↓)	↓↓	=	(↓)	=	=
S2pPol II	gene body	ND		↓↓	=	↓↓	=	↓↓	↓↓	=	=
ERCC3	GAS	ND		ND		=	↓↓	ND		ND	
CDK9	GAS	ND		ND		=	↓↓	ND		ND	
MED18	GAS	ND		ND		=	=	ND		ND	
MED4	GAS	ND		ND		↓↓	=	ND		ND	
MED24	GAS	ND		ND		(↓)	↓↓	=	↓↓	ND	
MED26	GAS	ND		ND		↓	↓↓	=	=	ND	
MED1	GAS	ND		ND		↓↓	↓↓	↓↓	=	ND	

Changes between Stat1^β/β^ and WT cells are indicated by symbols: =, no change; –, not induced by IFNγ and no change; ↑, increased; ↓, decreased; ↓↓, strongly decreased (i.e., more than 2-fold) with p ≤ 0.05; (↓), decreased with a p value between 0.05 and 0.1 in Stat1^β/β^ compared to WT cells; NA, not applicable; ND, not determined

a previously published data (25)

b *increased at 24 h after treatment*.

The most important finding of our study is that the STAT1 C-terminal TAD is required for an efficient association of components of the Mediator complex to the *Irf1* and *Irf8* promoters and an efficient release of poised Pol II. Many TFs interact directly with the Mediator complex, although TFs target distinct Mediator subunits (66). With the exception of STAT2, it is unclear how STAT proteins interact with the Mediator complex. STAT2 binding to MED14 increases ISGF3-induced transcription but it remained undetermined whether the contact to MED14 is through the C-terminal TAD of STAT2 (65). Our data indicate that the STAT1 C-terminal TAD is involved in the recruitment of components of the Mediator tail (MED24), middle (MED4) and flexible (MED1, MED26) submodules to the STAT1 homodimer-driven *Irf1* gene. Pol II recruitment was not affected by the absence of the STAT1 C-terminal TAD indicating that Pol II binding to the *Irf1* promoter is independent of the core Mediator complex which, according to the definition as the minimal set of Mediator subunits required to reconstitute a functional Mediator complex *in vitro*, consists of head and middle modules held together by MED14 (67). Interestingly, we show that the recruitment of MED18, a component of the Mediator head submodule, to the *Irf1* promoter does not require the presence of the STAT1 C-terminal TAD. This is in line with the current concept that the head module of the Mediator complex interacts with Pol II (67) and suggests that this does not require input from the STAT1 C-terminal TAD. Our data are also consistent with a previous study that indicated impaired recruitment of MED1 to the *Irf1* promoter in the absence of STAT1α (18). Notably, the STAT1 S727A mutation did not affect *Irf1* transcription (18), arguing against the requirement for S727 phosphorylation for the recruitment of the Mediator core complex to the *Irf1* gene.

Another interesting finding of our study is that the STAT1 C-terminal TAD facilitates the association of TFIIH and p-TEFb to the *Irf1* promoter in a time-dependent manner, as evidenced by the promoter occupancy of the TFIIH component ERCC3 and the p-TEFb kinase CDK9. Within the first hour of IFNγ treatment, promoter occupancy of ERCC3 and CDK9 did not differ between *Stat1*^β/β^ and *WT* cells, suggesting that the recruitment of these GTFs to the *Irf1* promoter is independent of the STAT1 C-terminal TAD and the Mediator core complex. In contrast, promoter occupancy of ERCC3 and CDK9 was strongly reduced in *Stat1*^β/β^ compared to WT cells at 6 h after treatment. While the reduced promoter occupancy of ERCC3 did not correlate with significant differences in the levels of S5 phosphorylated Pol II, promoter occupancy of S2 phosphorylated Pol II at the *Irf1* TSS was clearly lower in *Stat1*^β/β^ than in *WT* cells, which is consistent with a role of CDK9 in the phosphorylation of S2 of Pol II. It has to be taken into consideration that *Irf1* transcription is induced within 30–60 min after IFNγ treatment (35, 45) and thus data at the 6 h time point may reflect effects on transcriptional re-initiation. Transcriptional re-initiation is facilitated by scaffold PICs that remain after Pol II escape, contain most of the pre-initiation factors, including TFIIH and Mediator, and are stabilized by TFs (3, 68). It thus seems reasonable to speculate that the STAT1 C-terminal TAD may be required to stabilize re-initiation scaffolds at the *Irf1* promoter. The STAT1 C-terminal TAD is also required for the recruitment of CDK8, a component of the Mediator kinase module, which has been implicated in multiple aspects of the transcriptional cycle, including transcriptional re-initiation (3). The recent finding that STAT1 requires processive transcription for its dephosphorylation and promoter dissociation (69) prompts the hypothesis that transcriptionally compromised STAT1β homodimers accumulate at the promoters and prevent transcriptional re-initiation. However, it is also possible that the time-dependent effects observed relate to the heterogeneity of the cell population and reflect an increase in the number of cells responding to IFNγ over time. Further studies are required to distinguish between these possibilities and to test a potential involvement of the STAT1 C-terminal TAD in the regulation of transcriptional re-initiation.

It also remains to be investigated how the STAT1 C-terminal TAD mediates the transition of poised Pol II at the *Irf1* promoter into productive elongation within the first hour of stimulation. TFIIH and S5 phosphorylation of Pol II were not affected by the absence of the STAT C-terminal TAD, indicating unimpaired early elongation. The release of paused Pol II into productive elongation requires phosphorylation of negative elongation factors by p-TEFb. The association of the p-TEFb kinase CDK9 with the *Irf1* promoter was not affected by the absence of the STAT1 C-terminal TAD at 1 h after stimulation, arguing against an impaired recruitment of p-TEFb as underlying mechanism. However, different p-TEFb-containing complexes may be recruited in the absence or presence of the C-terminal TAD (67, 70). Another interesting possibility is that the absence of the C-terminal TAD might result in premature transcriptional termination due to a failure to recruit MCM5-containing complexes. This hypothesis is supported by previous studies that demonstrated interactions of MCM5 with the STAT1 C-terminal TAD (17) and IFNγ-induced association of MCM5 and MCM3 with the promoter and intergenic regions of *Irf1* (71), suggesting that MCM2-MCM7 complexes move along with Pol II during *Irf1* transcript elongation possibly unwinding DNA through their helicase activity (71).

Importantly, the STAT1 C-terminal TAD facilitates, but is not absolutely required, for the recruitment of Mediator components to the *Irf1* and *Irf8* promoters and for its transcriptional activity at these genes. This is in line with earlier studies demonstrating that STAT1β is capable of inducing transcription of naked DNA in transcription assays *in vitro* (21). In contrast to our study, cell transfection experiments indicated an absolute requirement for the STAT1 C-terminal TAD for the induction of *Irf1* (13, 21). The reason for this discrepancy is unclear, but may relate to the presence of paused Pol II and active histone marks at the *Irf1* locus in primary macrophages (58). In the fibrosarcoma cell line 2fTGH, the *Irf1* gene requires STAT1-dependent histone methylation, including H3K4me3, for its transcriptional induction by IFNγ (72) whereas we and others (58) show that H3K4me3 is already high under basal conditions and does not further increase upon IFNγ or lipopolysaccharide treatment in primary macrophages. Notably, the *Irf1* promoter also has active chromatin marks in many primary human cell types, including cells of the myeloid lineage (73).

In contrast to *Irf1* and *Irf8*, induction of the *CIIta* gene was completely abolished in *Stat1*^β/β^ cells. Unresponsiveness to STAT1β correlated with an impaired IFNγ-induced histone acetylation (H3ac and H4ac) and H3K4me3 at the *CIIta* promoter and a failure to recruit Pol II. In line with the ChIP data, IFNγ-induced *CIIta* pre-mRNA synthesis and up-regulation of MHC class II proteins at the cell surface were completely abolished in the absence of the STAT1 C-terminal TAD. As *Stat1*^β/β^ cells show a considerably reduced upregulation of IRF1, we cannot distinguish whether the impaired induction of *CIIta* is due to a role of the STAT1 C-terminal TAD at the *CIIta* promoter or to the reduced availability and promoter occupancy of IRF1.

In contrast to *CIIta* pIV, H3, and H4 acetylation at the *Gbp2* promoter was not dependent on the C-terminal TAD of STAT1. This is surprising, as previous studies using *Stat1*^−/−^ and *Irf1*^−/−^ cells suggested that H4 acetylation at the *Gbp2* promoter is mediated through STAT1, although these studies are complicated by the fact that *Irf1*^−/−^ cells have reduced STAT1 protein levels and *Stat1*^−/−^ cells fail to upregulate IRF1 (45). Further support for an involvement of STAT1 in the recruitment of histone acetyltransferases (HATs) to the *Gbp2* promoter came from the analysis of cells harboring a point mutation of S727 within the C-terminal TAD (*Stat1*^*S*727*A*^), which have strongly reduced H4 acetylation and fail to recruit CBP to the *Gbp2* promoter (19, 45). The reason for the discrepancy between *Stat1*^β/β^ and *Stat1*^*S*727*A*^ remains unclear. It seems possible that STAT1 recruits HATs directly or indirectly through regions distinct from the C-terminal TAD (24) and that this is inhibited by S727 phosphorylation of the TAD. Alternatively, the absence of the C-terminal TAD and mutation of S727 may differentially affect recruitment of HATs and histone deacetylases (HDACs). Further studies are required to delineate the exact role of the STAT1 C-terminal TAD and its serine phosphorylation in the recruitment of HATs and HDACs and acetylation of H3 and H4 at specific lysine residues. Despite the unimpaired histone acetylation, recruitment of Pol II and transcriptional induction of *Gbp2* were severely impaired in the absence of the STAT1 C-terminal TAD, indicating that histone acetylation is not sufficient to recruit Pol II and induce gene expression. However, low-level of Pol II recruitment still occurred in *Stat1*^β/β^ cells which correlated with impaired, but not absent, transcriptional activity and may relate to the interaction of IRF1 with Pol II (45).

Collectively, our data provide the first evidence that the STAT1 C-terminal TAD facilitates transcription through the recruitment of the Mediator complex to GAS-driven genes that harbor an open chromatin state. Our study also provides further evidence for the hypothesis that regions distinct from the C-terminal TAD contribute to the transactivating activity of STAT1. It remains to be investigated whether the gene-specific requirement for the STAT1 C-terminal TAD for histone acetylation at GAS-driven genes reflects gene-specific functional cooperativity with other TFs or co-factors or the recruitment of distinct HATs or HDACs. It also has to be taken into consideration that STAT1 activity at distal enhancers may contribute to the gene-specific transactivating activity of STAT1β (48, 74–76). Many aspects of innate and adaptive immunity are regulated by STAT1. Thus, a better understanding of its interaction with the transcriptional machinery and of the function of its individual isoforms may help to fine-tune therapeutic and diagnostic strategies that interfere with STAT1 functions.

## Author Contributions

MP performed most of the experiments. KM did the laboratory work related to the FACS experiments, contributed to data interpretation, performed the statistical analysis, helped with data presentation, and edited the manuscript. MO and TL performed qPCR analyses, AP performed some of the ChIP experiments and SW provided help with the ChIP technology. PK, TD, and MM were involved in the study design, provided crucial input throughout the project, and edited the manuscript. BS designed the study and wrote the manuscript. MM critically reviewed the manuscript. BS and MM obtained funding. All the authors approved the manuscript.

### Conflict of Interest Statement

The authors declare that the research was conducted in the absence of any commercial or financial relationships that could be construed as a potential conflict of interest.
