# Supplementary material for: The C-Terminal Transactivation Domain of STAT1 Has a Gene-Specific Role in Transactivation and Cofactor Recruitment
Source: Front Immunol. 2018 Dec 6;9:2879. doi: 10.3389/fimmu.2018.02879 (PMC6291510; doi:10.3389/fimmu.2018.02879)
Supplement: Supplementary file 1 [file Data_Sheet_1.docx]

Supplementary Material

The C-terminal Transactivation Domain of STAT1 Has a Gene-specific Role in Transactivation and Cofactor Recruitment

Matthias Parrini, Katrin Meissl, Mojoyinola Joanna Ola, Therese Lederer, Ana Puga, Sebastian Wienerroither, Pavel Kovarik, Thomas Decker, Mathias Müller and Birgit Strobl^*^

*** Correspondence:** Birgit Strobl: [birgit.strobl@vetmeduni.ac.at](mailto:birgit.strobl@vetmeduni.ac.at)

# Supplementary Figures and Tables

**Supplementary Table 1.** List of primers used for pre-mRNA analysis. Intron-exon locations of primers are given in brackets.

|  | forward | reverse |
| --- | --- | --- |
| *Irf1* pre-mRNA (Exon 9-Intron 9/10) | ACATCGATGGCAAGGGATAC | GCATGCTGGGATGCTTTAAT |
| *Irf7* pre-mRNA (Intron 7/8-Exon 8) | CTGCCCTACTGCTCCTTCAC | GATGGTCACATCCAGGAACC |
| *Irf8* pre-mRNA (Intron 7/8-Exon 8) | TACACACAGCTCAGCGGTTC | ACCACCCTGCTGTCAGGTAG |
| *CIIta* pre-mRNA (Intron 7/8-Exon 8) | CCCAGCACACTTCTGGATG | GAAGGGAGCACTTGGTTGAC |
| *Gbp2* pre-mRNA (Intron 4/5) | ACAGCATCATTATTACATCAGG | ATACCGAGCCAGATAGGG |
| *Ube2d2* pre-mRNA (Exon 6-Intron 6/7) | TTGTGTGATCCCAATCCAGA | AGCTCTATCCCATGCCTGAA |

**Supplementary Table 2.** List of primers used for ChIP analysis. Intron/exon locations of the primers used for the analysis of gene bodies are given in brackets.

|  | forward | reverse |
| --- | --- | --- |
| *Irf1* GAS | GGAGCACAGCTGCCTTGTACTT | CCCACTCGGCCTCATCATT |
| *Irf1* TSS | TCCCGCTAAGTGTTTAGATTTC | TTCGGTTCGGCTTAGACTG |
| *Irf1* gene body (Intron 8/9) | TGCCTAGTTGCTTGTCTCTG | CTCCTGTGTGTCGCTGTC |
| *Irf7* ISRE | TGGTAGGCATGGAGACAGTG | AAACGAAACTGCATCTCAGGA |
| *Irf7* TSS | GCTCCGGTACCAGGTATGTG | GCTTCTCTTCACTGCCCTTC |
| *Irf7* gene body (Intron 7/8) | GCTCCTTCACACCTGCATCT | GGTAGGTTTGGGTGGAGCAT |
| *Irf8* promoter | TCCTTGACCTTAGGCAGACG | TCCGAGAAATCACTTTTGCAC |
| *Irf8* TSS | CTGTGGCTCTCCCACCTTC | GGAGAGCAGCCAGAGCAAG |
| *Irf8* gene body (Exon 9) | AAGACCCATTCACTGGCATC | CAGAGCACAGTTTGGACGTG |
| *Gbp2* GAS/ISRE | TGGTGCTAAAATTGTTGTGGGA | AGTGAAAGTGAATCTGAGTTCCT |
| *Gbp2* ISRE | TCTACCTGAGAAGTCCTGAG | TTGCCAGAGAACTTGTGAG |
| *Gbp2* TSS | CCCGGGTTACTACAGGGTCT | CTGGTCAGCTTTGCCTCTG |
| *Gbp2* gene body (Intron 4/5) | ACAGCATCATTATTACATCAGG | ATACCGAGCCAGATAGGG |
| *CIIta* GAS | GCTTCTGAGAAAGCACGTGG | TGTCTACACCTTCAGCTTTGGG |
| *CIIta* IRF-E | CCCAAAGCTGAAGGTGTAGACA | AGTCTCCTGGCAGCTATCTCA |
| *CIIta* TSS | TGTCTCCAAGATCCCCTTTG | ATAGTTGCCAGCCTCAGAGC |

**Supplementary Figure 1.** Original blots (Chemidoc scans) related to **Figure 1E**. BMDMs from *WT*, *Stat1^β/β^* and *Irf1^-/-^* mice were stimulated with IFNγ for the times indicated or left untreated (-). Protein extracts were isolated and **(A)** IRF1 protein levels were determined by Western blotting. **(B)** ERK p42 was used as loading control. Data are as representative of two independent experiments.

**Supplementary Figure 2.** Original blots (Chemidoc scans) related to **Figure 4A**. BMDMs derived from *WT*, *Stat1^β/β^, Stat2^-/-^,* *Irf9^-/-^,* *Stat1^β/β^Stat2^-/-^* and *Stat1^β/β^Irf9^-/-^* mice were stimulated with IFNγ for the times indicated or left untreated (-). Protein extracts were isolated and **(A)** Tyr701-phosphorylated STAT1 (pSTAT1) and **(B)** STAT1 protein levels determined by Western blotting. **(C)** ERK p42 was used as loading control. One representative out of three independent experiments is shown. For simplicity, *Stat1^β/β^, Stat1^β/β^Stat2^-/-^* and *Stat1^β/β^Irf9^-/-^* data were cropped and shown in Figure 4A.
